# Fecal Immunochemical Test and Multitarget Stool DNA Testing for Colorectal Cancer Screening in Real-World Practice: A Literature Review

**DOI:** 10.3390/jcm15114219

**Published:** 2026-05-29

**Authors:** Ashish Sharma, Angad Tiwari, Ishita Ray, Ruchir Paladiya, Harendra Kumar, Sukhmani Sidhu, Saloni Haldule, Hareesha Rishab Bharadwaj, Saqr Alsakarneh, Manesh Kumar Gangwani, Hassam Ali, Dushyant Singh Dahiya

**Affiliations:** 1 Department of Internal Medicine, Yale New Haven Hospital, New Haven, CT 06510, USA; ashish.sha688@gmail.com; 2 Department of Internal Medicine, Maharani Laxmi Bai Medical College, Jhansi 284128, Uttar Pradesh, India; 3 Department of Internal Medicine, Mahatma Gandhi Memorial Medical College, Indore 452001, Madhya Pradesh, India; ishitaraymd@gmail.com; 4 Department of Internal Medicine, University of Connecticut School of Medicine, Hartford, CT 06106, USA; 5 Mayo Clinic, Rochester, MN 55905, USA; kumar.harendra@mayo.edu (H.K.);; 6 Department of Internal Medicine, Royal Stoke University Hospital, University Hospitals of North Midlands NHS, Stoke-on-Trent ST4 6QG, UK; rishabbharadwaj31@gmail.com; 7 Department of Gastroenterology and Hepatology, University of Arkansas For Medical Sciences, Little Rock, AR 72205, USA; 8 Department of Gastroenterology, Hepatology and Nutrition, Brody School of Medicine, East Carolina University, Greenville, NC 27834, USA; 9 Division of Gastroenterology, Hepatology & Motility, The University of Kansas School of Medicine, Kansas City, KS 66160, USA

**Keywords:** colorectal cancer, fecal immunochemical test (FIT), multitarget stool DNA test (mt-sDNA), preventive oncology

## Abstract

Colorectal cancer (CRC) is responsible for a high cancer burden and a high number of deaths all over the world, although effective screening can make it preventable to a significant extent. Stool-based tests, such as the fecal immunochemical test (FIT) and multitarget stool DNA (mt-sDNA) testing, are gaining considerable popularity as non-invasive procedures that can be a replacement for colonoscopies for people at an average risk for colon cancer. Despite evidence from several randomized controlled trials supporting the use of these tests for colorectal cancer screening, their external validity in a real-world setting is influenced by many factors such as adherence, timely follow-up post testing, the healthcare cost burden, accessibility and the capacity of the health system. In this article, we have performed an extensive narrative literature review of research published between 2020 and 2025 comparing FIT and the mt-sDNA test with reference to diagnostic accuracy, cost-effectiveness, adherence and outcomes of implementation. We discuss the issues of sensitivity and specificity, look at post-test requirements for colonoscopy and check if there is any discrimination in healthcare. These findings suggest that FIT and mt-sDNA tests should not be considered competing technologies but rather complementary screening methods, with their overall effectiveness contingent upon appropriate patient selection and widespread system-level implementation. It is crucial to combine strategic test selection with a robust follow-up infrastructure to ensure that the entire population benefits from the CRC prevention program.

## 1. Introduction

Colorectal cancer (CRC) is a major global public health concern and one of the leading causes of cancer-related mortality worldwide, alongside lung and breast cancers [1,2]. GLOBOCAN 2023 projections estimate over 1.9 million new cases and more than 900,000 deaths annually, making CRC the second most common cause of cancer death globally [3]. The burden is increasing in low- and middle-income countries due to dietary transitions, reduced physical activity, and limited implementation of screening programs [4]. Conversely, organized screening initiatives in the United States, Canada, and parts of Europe have contributed to significant reductions in CRC incidence and mortality over the past two decades [5].

Despite this progress, screening uptake remains suboptimal. Approximately one-third of eligible adults in the United States do not undergo recommended screening, with disparities disproportionately affecting minority, underserved, and rural populations [5,6,7,8]. Barriers include fear of cancer, lack of awareness, logistical challenges, and limited trust in healthcare systems [9], leading to delayed diagnosis and worse outcomes. Colonoscopy is considered the gold standard for CRC screening due to its high sensitivity and ability to perform therapeutic interventions such as polypectomy [10]. However, it is invasive, resource-intensive, requires bowel preparation and sedation, and often necessitates time away from work, which reduces patient compliance. Additionally, access to colonoscopy is limited by healthcare infrastructure and workforce constraints, particularly in resource-limited settings [10,11,12,13,14,15,16].

**Figure 1:**
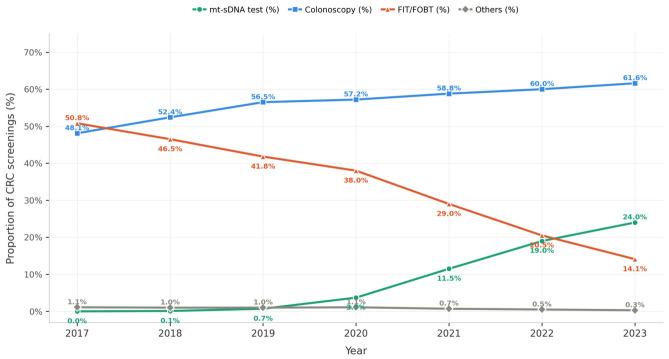
Trends in colorectal cancer screening modality utilization among insured individuals in the United States, 2017–2023. Proportions represent the percentage of all CRC screenings performed per year. Data source: Fisher, D.A., et al. (2021); Balcerak, G., et al. (2020) [36,63]. Abbreviations: CRC, colorectal cancer; FIT, fecal immunochemical test; FOBT, fecal occult blood test; mt-sDNA, multitarget stool DNA testing.

As a result, stool-based screening tests have emerged as practical, non-invasive alternatives for average-risk individuals [17,18]. Among these, the fecal immunochemical test (FIT) and multitarget stool DNA (mt-sDNA) testing are widely utilized and recommended by major guidelines [18] (Figure 1). These tests differ in their mechanisms, screening intervals, and implementation characteristics, and their effectiveness in real-world practice is influenced by factors such as adherence, follow-up completion, healthcare costs, and system capacity. This review synthesizes literature published between 2020 and 2025 comparing FIT and mt-sDNA testing in real-world settings, focusing on diagnostic performance, cost-effectiveness, adherence, and health system implications to inform patient-centered and system-level screening strategies.

## 2. Methodology

This narrative literature review was conducted to synthesize and critically evaluate the comparative real-world evidence on FIT and mt-sDNA testing. A thorough literature search was carried out that included all relevant articles identified as studies of different aspects of the FIT and mt-sDNA tests in CRC screening in real-world settings. The methodology of the literature search included searching major databases like PubMed/MEDLINE, Embase, Scopus, and Cochrane Library and searching ClinicalTrials.gov for both ongoing and completed trials. Key article reference lists were also screened for completeness.

The following keywords and Medical Subject Headings (MeSH) were used in various combinations:

- “Fecal immunochemical test” OR “FIT” OR “occult blood test”;
- “Multitarget stool DNA” OR “mt-sDNA” OR “mt-sDNA” OR “stool DNA testing”;
- “Colorectal cancer screening” OR “CRC screening” OR “colon cancer prevention”;
- “Diagnostic accuracy”, “cost-effectiveness”, “adherence”, “real-world evidence”, “follow-up outcomes”.

Boolean Operators (i.e., AND/OR) helped in scaling down search results. All searches were performed within the period between January 2020 and December 2025, which provided recent reports on clinical trials, implementation studies and real-world evidence. Only articles that were written in English and published in a satisfactory way (peer-reviewed articles, clinical guidelines, trial reports) were considered for inclusion.

Articles were selected by title and abstract to filter out non-eligible articles, and subsequently, the full-text articles were reviewed. Information obtained on qualified studies pertained to study design, patient population, intervention (e.g., frequency, protocols of tests), outcome measured (e.g., sensitivity, specificity, adherence rates, cost implications), and implementation problem (e.g., health system burden and equity issues). Randomized controlled trials were included, as well as real-world observational evidence, to provide a comprehensive overview.

## 3. Overview of Stool-Based Screening Tests

### 3.1. Fecal Immunochemical Test (FIT)

The FIT is an assay that detects stool occult blood through the use of antibodies that are extremely specific for the globin part of human hemoglobin, hence making blood-loss detection in the lower gastrointestinal tract more accurate [14]. However, guaiac-based fecal occult blood tests (gFOBTs) require strict dietary and medication restrictions because the test is based on a peroxidase reaction; heme from red meat, peroxidase activity in certain vegetables and some medications can interfere with the assay and lead to inaccurate results; however, this is not the case with FIT [19,20,21]. This increases patient acceptability and adherence, diminishing pre-analytical variability. Thus, FIT is regarded as the gold standard in non-invasive screening practices in the current clinical scenario [18].

FIT is usually performed on a single stool sample and offers quantitative results, which means that the healthcare systems can set different positivity thresholds depending on the risk profiles of their population and the capacity for colonoscopy [22]. This adaptability has made it possible to use different screening methods according to the risk level in various organized screening programs, especially in Europe and North America [18,23]. Annual FIT screening for average-risk adults aged 45–75 years is supported by most international guidelines, including those from the U.S. Preventive Services Task Force and the American Cancer Society, as the procedure presents a favorable ratio of effectiveness, safety and cost [15].

In the eyes of the health system, FIT is a low-cost, scalable, and smoothly integrated method into systematic screening programs, especially those based on mail outreach combined with centralized laboratory processing. Population-based research has shown that the implementation of mailed FIT programs leads to a significant rise in screening participation, especially in marginalized communities and people without regular primary care access. FIT screening has been reported to be linked to the decline of CRC incidence and mortality when conducted over a period of time consistently [5,24,25].

Yet FIT’s effectiveness hinges majorly on its repeated annual participation, which is evidently difficult to implement in the real world. Several longitudinal studies have found that the confirmation of adherence to annual FIT goes down substantially after the first screening cycle, with some areas reporting repeat participation rates already below 50% after two to three years [26]. However, these rates reflect heterogeneous implementation contexts and should not be interpreted as inherent to FIT-based screening, as organized programs with robust recall infrastructure have achieved substantially higher sustained adherence. Reasons for reduced adherence are screening fatigue, absence of reminder systems, other health issues taking precedence and limited patient awareness about the necessity of repeated testing [27,28]. If there are no effective recall systems and patient navigation at hand, the long-term preventive effect of the FIT-based screening programs will be greatly diminished [29,30,31].

### 3.2. Multitarget Stool DNA Testing (mt-sDNA)

mt-sDNA testing is a cutting-edge technique for non-invasive screening of CRC that detects not only occult blood but also several molecular markers related to the presence of colorectal neoplasia.

The test detects CRC–associated changes by examining stool samples for specific DNA methylation markers (such as NDRG4 and BMP3), KRAS gene mutations and β-actin as an internal reference to assess the adequacy of the sample to the pairing of molecular and immunochemical techniques. A wider range of detection is achieved than in the case of traditional fecal tests, which detect only lesions that are not yet bleeding [32,33].

mt-sDNA testing is advisable for average-risk adults aged 45 to 75 at three-year intervals, which is consistent with the screening recommendations of the U.S. Preventive Services Task Force (USPSTF) and the American Cancer Society [34,35]. In the critically important DeeP-C trial, mt-sDNA revealed a 92.3% sensitivity for CRC and a 42.4% sensitivity for advanced adenomas, making it significantly better than FIT in both detection categories [36,37]. It should be noted that the DeeP-C trial employed a cross-sectional case-enriched design rather than a population-based screening RCT, which may inflate sensitivity estimates relative to real-world screening performance. Direct comparisons between mt-sDNA and FIT sensitivity figures should therefore be interpreted with this in mind. Real-world studies within integrated healthcare systems have corroborated the above findings, demonstrating high sensitivity with single-test use in some cases and improved screening compliance among individuals who were previously unscreened [38,39]. An important benefit of mt-sDNA testing is its wide acceptance by the patient population, especially by those hesitant to undergo a colonoscopy or to be screened with an annual FIT. People who participated in survey-based studies have indicated that they find mt-sDNA screening to be more convenient and less invasive, making it easier to overcome the usual psychosocial obstacles to screening [22,40,41]. However, the mt-sDNA examination costs remarkably more, generally $500–600 per test, compared to less than $30 for FIT. Additionally, the specificity (86–90%) is lower than that of FIT (94–97%), leading to a higher false-positive rate and culminating in unnecessary invasive colonoscopies for confirmatory testing [42,43]. This can potentially create a situation of increased follow-up, overwhelming endoscopy resources and contributing to heightened patient anxiety and increased procedural risk. The logistics of administering the mt-sDNA test are more complicated than those for FIT [44,45]. mt-sDNA kits are sent directly to patients, and samples are taken and sent back by the patients themselves within a certain time window. Errors in handling, delays or missed sample collection can invalidate the test and diminish the effectiveness of the screening process [46]. Nevertheless, mt-sDNA has shown great results in organized outreach models that involve care coordination and patient navigation [18]. Although mt-sDNA testing is not meant for regular screening, its higher one-time detection capacity makes it a desirable option in cases where there are populations with poor repeat adherence or limited engagement with the healthcare system [18,33]. Further research is required to assess cost-effectiveness, determine optimal implementation intervals and evaluate equity-related outcomes across diverse settings [47].

### 3.3. Guideline Recommendations

FIT and mt-sDNA testing have been recognized as non-invasive methods for CRC screening in average-risk adults by major international and national guideline-issuing organizations. The USPSTF, in its 2021 updated recommendations, has included both tests in its list of preferred tests for 45- to 75-year-old individuals, citing evidence in support of their effectiveness in reducing CRC mortality [32]. The American Cancer Society (ACS) has similarly indicated that either annual FIT or triennial mt-sDNA testing should be a part of a menu of options, stressing that early detection strategies must be patient-centered and consider the capacity of the system as well [18].

Guidelines are increasingly acknowledging that test results alone should not determine test selection. Instead, the “best test” is the one a patient is most likely to complete, especially when a positive result will necessitate a follow-up colonoscopy [18]. This is a new patient-oriented approach based on real-world evidence that shows that completion and adherence to the screening test are as important as sensitivity in determining the impact on the population [48]. The National Comprehensive Cancer Network (NCCN) and other specialized medical societies have supported this viewpoint, insisting on the necessity of considering patient preferences, test access issues and cultural factors when making decisions about screening guidelines [36,49]. Moreover, the growing emphasis on shared decision-making in primary care and public health highlights the significance of offering multiple screening options to patients. Studies have shown that allowing patients to choose among FIT, mt-sDNA or colonoscopy significantly increases screening uptake, notably among individuals who have not previously undergone screening [18]. Before, guideline development valued science in addition to approaches that are challenging to implement yet beneficial for society, patient rights and the long-term sustainability of the healthcare system.

## 4. Diagnostic Performance: Interpreting the Numbers

The screening test for CRC diagnosis mainly depends on its sensitivity, specificity and capability to find not just early-stage cancers but also advanced adenomas, which are the main precursors to invasive malignancy. FIT and multitarget stool DNA (mt-sDNA) testing are both capable of recognizing colorectal neoplasia. However, the test characteristics differ considerably between both testing methods, which ultimately affects their real-world clinical usability.

### 4.1. Sensitivity for Colorectal Cancer

**Figure 2:**
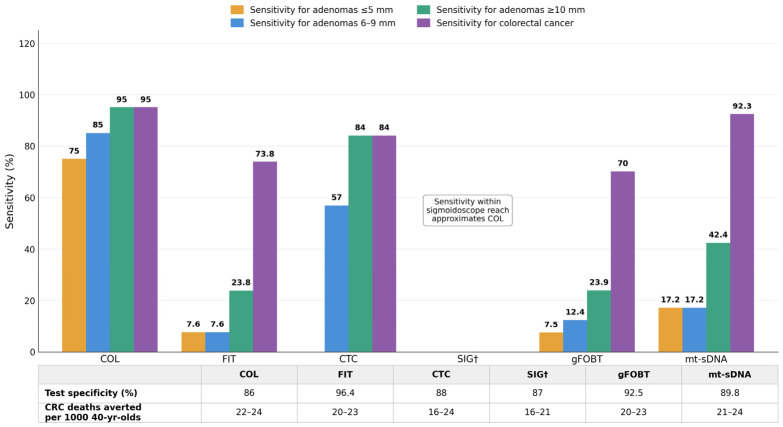
Comparative sensitivity of colorectal cancer screening modalities by lesion size and cancer, with test specificity and estimated colorectal cancer deaths averted per 1000 40-year-olds. Data derived from: Knudsen, A.B., et al. (2016) and Imperiale, T.F., et al. (2014) [32,64]. Abbreviations: COL, colonoscopy; CTC, computed tomographic colonography; FIT, fecal immunochemical test; gFOBT, guaiac-based fecal occult blood test; mt-sDNA, multitarget stool DNA testing; SIG, sigmoidoscopy; FIT-DNA, fecal immunochemical test-DNA (alternative term for mt-sDNA); CRC, colorectal cancer.

Sensitivity describes a test’s capacity to correctly detect the presence of a condition in patients. Numerous studies and meta-analyses published from 2020 onwards have shown that the sensitivity of mt-sDNA testing is significantly higher than that of FIT for the detection of CRC in all cases (Figure 2). A systematic review by Robertson et al. [30] indicated that mt-sDNA testing achieved a pooled sensitivity of 92.3% for CRC versus 74.0% for FIT. The results of the DeeP-C trial, a large multicenter study, also corroborated these findings, as it showed that the sensitivity for mt-sDNA was 92.3% and for FIT was 73.8% when tested in more than 10,000 average-risk individuals [50].

Recent analyses performed in real-world settings have supported these results. For instance, a study based on Medicare claims in 2022 demonstrated that mt-sDNA testing detected nearly twice as many CRC cases per 1000 screened individuals within one year compared to FIT in a matched cohort of more than 50,000 participants [51]. Likewise, the data collected from large healthcare systems like Kaiser Permanente supported mt-sDNA as the source of more efficient early-stage cancer detection, especially for younger patients aged less than 60 [51,52]. The variation in sensitivity is very stark when we screen patients for early-stage CRC, characterized by either intermittent or no bleeding. mt-sDNA tests uncover molecular changes (e.g., KRAS mutations and hypermethylated promoter regions) and therefore, they are capable of identifying neoplastic lesions prior to the onset of occult bleeding, thereby offering an advantage over FIT, which relies solely on the detection of hemoglobin [18,31]. Furthermore, while single-round sensitivity favors mt-sDNA, the cumulative detection rate of annual FIT over multiple screening rounds progressively narrows this gap, an important consideration when evaluating real-world program effectiveness.

### 4.2. Advanced Adenoma Detection

The detection of advanced adenoma precursors with a high risk of malignant transformation to cancer is an important performance metric, as their removal leads to a considerable reduction in future CRC risk. All stool-based tests continue to have limited sensitivity for detecting advanced adenomas, which is a significant limitation. The mt-sDNA test produced higher sensitivities for advanced adenomas compared to FIT in comparative trials; however, neither of them could reach the diagnostic performance of a colonoscopy. The DeeP-C study reported sensitivities of 42.4% for mt-sDNA and 23.8% for FIT in their performance in detecting advanced adenomas [30,53]. More recent evidence from the National Health Interview Survey and Veterans Affairs cohorts indicates sensitivity ranges of approximately 40–50% for mt-sDNA and 20–40% for FIT, based on polyp size and histologic features. The sensitivity of mt-sDNA improves significantly as adenoma size increases. For instance, it has been published that the sensitivity for lesions larger than 2 cm is about 60%, and it is less than 30% for smaller lesions [18]. This not only highlights the possible use of mt-sDNA in the detection of higher-risk precursors but also puts emphasis on the indispensable role of repeating the test and performing surveillance colonoscopy as a part of the overall cancer prevention strategy throughout the years [54].

### 4.3. Specificity and False Positives

In general, specificity denotes the degree of a test’s efficacy in detecting the population that is disease-free correctly. The evidence does support the fact that FIT gives consistently better results as far as specificity is concerned when compared to the mt-sDNA test. Most of the large-scale trials and studies place the FIT specificity in the range of 94–97% specificity, while mt-sDNA has a specificity in the range of 86–90%, consequently resulting in a lesser number of false positives in the case of FIT [13,18]. The implications of this variation in specificity are quite significant. A higher specificity of mt-sDNA testing implies that a greater number of follow-ups, i.e., colonoscopies, will be needed, and most of these will be negative. For instance, in a 2021 commercial insurance data analysis, about 16% of mt-sDNA patients were sent for a colonoscopy within six months of testing, compared to just 8% of FIT patients, with 40% of the mt-sDNA-positive colonoscopies revealing no pathology problems [34]. Although false positives lead to futile resource usage and anxiety for the patient, the argument in favor of the mt-sDNA test is that there is still benefit for populations that do not adhere well to annual testing [35]. However, counter-arguments highlight the cost and risk of carrying out unnecessary colonoscopies with limited specificity regarding high-grade lesions, particularly in low-risk groups [18]. When comparing sensitivity versus specificity, it is essential to note that higher sensitivity does not guarantee better outcomes for the population if accompanied by low specificity, low adherence or inadequate follow-up [18]. This principle deserves particular emphasis: simulation studies consistently demonstrate that a moderately sensitive test delivered with high adherence within an organized program may yield greater population-level cancer prevention than a highly sensitive test accompanied by poor follow-up or low repeat participation [55].

### 4.4. Risk Stratification and Emerging Biomarkers

The incorporation of risk stratification algorithms into the FIT process is an upcoming research avenue that intends to increase detection rates by modifying the hemoglobin cutoffs according to age, sex or comorbidity [31,56,57]. This method, already adopted in some European screening programs, has increased the detection rates, keeping colonoscopy demands at a resource-efficient level [57,58]. At the same time, other next-generation stool DNA assays are being developed, which will aid in the detection of mt-sDNA by introducing new methylation markers, microRNAs or fragment genomics [18,27,56,59]. Early-stage testing of these upcoming tests has revealed positive results, with some reporting sensitivity over 95% and specificity of 90%; however, there is a requirement for large-scale validation studies [18,60,61].

### 4.5. Cumulative Detection over Screening Cycles

An important consideration when comparing FIT and mt-sDNA is that the two tests operate on different screening intervals: annual for FIT versus triennial for mt-sDNA. This difference has substantial implications for cumulative detection rates and false-positive probabilities over equivalent time periods.

While mt-sDNA demonstrates higher single-round sensitivity for CRC (92.3% vs. 73.8% for FIT), the cumulative sensitivity of three annual FIT rounds over a 3-year period narrows this gap considerably. Modeling studies have estimated that the cumulative probability of detecting CRC over three consecutive annual FIT screenings ranges from 95% to 97%, accounting for incident cancers that develop between screening rounds, based on published single-round sensitivity estimates. This cumulative detection rate approaches or exceeds that of a single mt-sDNA test administered at the beginning of the same 3-year period.

Conversely, the cumulative false-positive probability must also be considered. With FIT specificity of 94–97%, the probability of at least one false-positive result over three annual rounds ranges from 9% to 17%, compared to the 10–14% false-positive rate of a single mt-sDNA test. This indicates that the per-round specificity advantage of FIT diminishes when cumulative exposure over multiple screening rounds is accounted for.

These cumulative effects underscore the importance of evaluating stool-based screening strategies on a program basis rather than a per-test basis. The choice between annual FIT and triennial mt-sDNA involves trade-offs not only in single-test performance but also in the frequency of patient contact, the temporal distribution of detection opportunities and the cumulative burden of follow-up procedures over time (Table 1).

**Table 1:** Diagnostic performance of FIT vs. mt-sDNA testing. Data derived from: Imperiale, T.F., et al. (2014); Robertson, D.J., et al. (2019); Chiu, H.M., et al. Gut. (2021) [18,32,62].

Test	CRC Sensitivity	Advanced Adenoma Sensitivity	Specificity	Screening Interval
FIT	70–85%	20–40%	94–97%	Annual
mt-sDNA	90–95%	40–50%	86–90%	Every 3 years

## 5. Cost, Resource Utilization, and Health System Impact

### 5.1. Test Cost and Reimbursement

The overall cost of screening is one of the major factors influencing healthcare decision-making, especially when it comes to large-scale population health interventions. The FIT is one of the lowest-cost CRC screening methods, with the average cost per test falling between $20 and $30 based on vendor contracts and laboratory setups [18]. However, the cost per mt-sDNA test is higher, at around $500–600, which is almost 20 times more than that of FIT [18,31].

When comparing costs over equivalent screening cycles, the difference narrows substantially. Over a 3-year period, the cost of three annual FITs is approximately $60–90 per patient, compared to $500–600 for a single mt-sDNA test. However, the total program cost must also account for the cumulative colonoscopy burden, which varies based on the cumulative false-positive rates discussed in Section 4.5.

In the United States, both tests are reimbursed by Medicare and most private insurance companies; however, payment policies can vary according to the number of tests performed and the requirements for diagnostic follow-up. For Medicare, mt-sDNA testing is covered once every three years for average-risk people aged 45–85, while the FIT is reimbursed once a year [22]. Nevertheless, in socioeconomically backward areas with low public health resources, the disparity in price at the start of screening might have an impact on patient choice [65].

**Figure 3:**
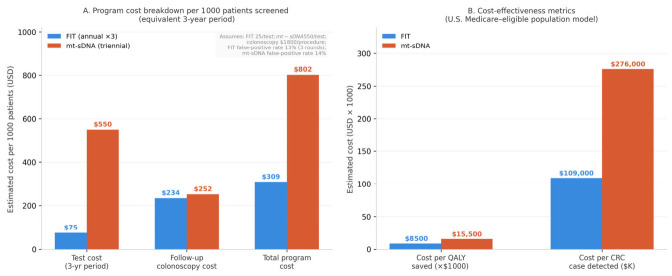
Estimated comparative cost analysis of FIT and mt-sDNA colorectal cancer screening programs. Panel (**A**): Estimated program costs per 1000 patients over an equivalent 3-year period, incorporating test cost and follow-up colonoscopy costs (assumptions: FIT $25/test ×3 annual tests; mt-sDNA $550/test ×1 triennial test; colonoscopy $1800/procedure; FIT false-positive rate 13% over 3 rounds; mt-sDNA false-positive rate 14%). Panel (**B**): Cost-effectiveness metrics including cost per QALY saved and cost per CRC case detected. Data derived from: Fendrick, A.M., et al. (2020); Knudsen, A.B., et al. (2016) [50,64]. Abbreviations: CRC, colorectal cancer; FIT, fecal immunochemical test; mt-sDNA, multitarget stool DNA testing; QALY, quality-adjusted life year.

A cohort-based modeling analysis by Fendrick et al. comparing FIT and mt-sDNA in a hypothetical average-risk U.S. population found that annual FIT resulted in approximately $3000 to $4000 lower cost per QALY compared to triennial mt-sDNA under conditions of optimal follow-up adherence [50] (Figure 3). However, it should be noted that QALY estimates derived from Markov models are highly sensitive to underlying assumptions, including adherence rates, follow-up colonoscopy completion and the disutility assigned to false-positive results. These assumptions were calibrated to a U.S. healthcare context and may not be generalizable to other settings, particularly healthcare systems outside the United States, where test pricing, colonoscopy capacity, reimbursement structures and population adherence patterns differ substantially. Cost-effectiveness conclusions from such models should therefore be interpreted as setting-specific rather than universally applicable [40,66]. It should also be noted that cost-effectiveness comparisons between FIT and mt-sDNA are most meaningful at the program level rather than on a per-test basis. When accounting for adherence rates, follow-up colonoscopy burden and detection across multiple screening rounds, the economic profile of each test differs substantially from what per-test cost figures suggest.

### 5.2. Colonoscopy Burden

One of the most significant downstream effects of stool-based screening is the requirement for colonoscopy services to follow up on positive results. Considering mt-sDNA’s lower specificity, there will be more false-positive results in a large fraction of the screened population, leading to the need for a diagnostic colonoscopy [18]. Data show that about 15–20% of positively testing mt-sDNA patients are referred for a colonoscopy, while the number is only 6–8% for FIT users in the same context [18]; of these referred patients, 35–45% do not have any advanced neoplasia found post colonoscopy, raising the issues of appropriate allocation of resources and healthcare cost burden on the patients [67].

The rise in superfluous colonoscopies can have several negative consequences, such as risks associated with the procedures (e.g., bleeding, perforation), an increase in people’s anxiety and additional avoidable healthcare costs. In addition, in places with limited financial resources, the excessive demand for follow-up could cause delays in performing diagnostic colonoscopy even for correctly diagnosed cases, thus possibly negating the clinical advantage of screening. Healthcare systems that do not have enough endoscopy facilities have to make a tough choice between the sensitivity and the demand for procedures that follow. Using mt-sDNA in such situations could lead to the creation of resource bottlenecks by drawing resources away from treating high-risk patients in more urgent need of these resources [18,67].

### 5.3. Health System Scalability

FIT’s low cost, operational simplicity, and seamless integration into mail-based workflows make it a highly scalable tool for use in organized screening programs. It has been implemented successfully in community health centers, federally qualified health clinics (FQHCs) and national cancer screening registries, frequently reaching high numbers of people at a low cost [68]. The Kaiser Permanente FIT-based outreach program and other large-scale projects have reported screening rates for CRC that are over 80% when the annual FIT is paired with automated reminders and navigation support [69]. However, mt-sDNA testing presents logistical issues that could limit its usage. Each kit for the test is made up of proprietary parts, and there is a necessity for secure shipping and handling as well as advanced laboratory analysis at the manufacturer’s centralized facility. The single-source processing model can lead to delays, less flexibility and susceptibility to supply chain interruptions, especially in remote, underserved areas [70,71].

In addition, patients are required to finalize the mt-sDNA collection process within a specified period and then quickly send the sample to the lab for results. These barriers are faced most by populations with poor transportation, limited internet access or low health literacy. Table 2 mentions operational requirements that render mt-sDNA less suitable for outreach-based or decentralized health delivery systems [72]. However, mt-sDNA could still serve as a useful alternative in places where person-to-person screening is difficult due to pandemics like COVID-19. mt-sDNA testing was used as a bridge screening tool during 2020–2021, when colonoscopy availability was limited, and some health systems even reported that mt-sDNA uptake had increased because of this. The selection of screening methods should be based not only on the efficiency of the tests but also on the preparedness of the system, population traits and the availability of resources. The models need to be scalable and cost-effective to incorporate the infrastructure for test delivery, tracking and follow-up to provide equitable coverage for CRC screening [33].

## 6. Adherence and Follow-Up Challenges

Stool-based CRC screening tests are effective not only according to how well the test performs but also by patient adherence and how many undergo a colonoscopy after a positive screening test. The real-world studies unvaryingly demonstrate that the lower the adherence, the greater the loss of benefit of the screening tools, even if they are the most sensitive ones.

### 6.1. Annual FIT Participation

**Figure 4:**
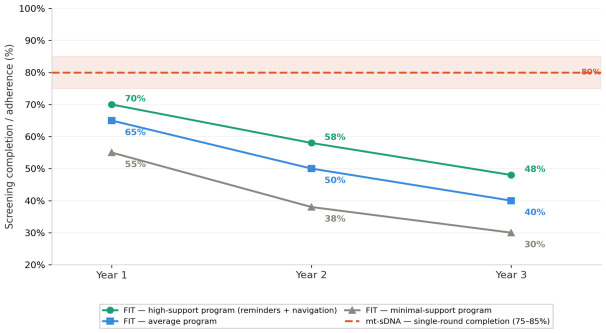
Longitudinal adherence to annual FIT screening stratified by implementation support level, compared with mt-sDNA single-round completion rate over a 3-year period. FIT adherence is shown for three program types: high-support (automated reminders, nurse navigation, multilingual outreach), average-support, and minimal-support. The mt-sDNA reference band (shaded) reflects the 75–85% first-round completion range reported across major studies. Data derived from: Halm, E.A., et al. (2025); Gupta, H., et al. (2025); Chubak, J., et al. (2022) [28,73,76]. Abbreviations: FIT, fecal immunochemical test; mt-sDNA, multitarget stool DNA testing.

The effectiveness of fecal immunochemical testing (FIT) depends majorly on uniform annual participation. Even though FIT is easy, inexpensive and user-friendly, actual compliance usually falls off quickly after the first test (Figure 4). The participation rate of residents in screening programs across the U.S., Canada and European countries is reported to decrease from 40% to 50% by the end of the third year of the program [73]. However, it is important to note that these figures vary substantially depending on the level of implementation support provided. Programs with minimal outreach infrastructure report adherence rates in the range of 30–40%, whereas those with intensive support including automated reminders, patient navigation and EMR-integrated recall systems have demonstrated sustained adherence rates exceeding 70–80% [69,73,74]. The reasons for declining adherence in poorly supported programs include screening fatigue, forgetfulness, lack of perceived urgency and absence of systematic reminder systems [49]. These findings suggest that FIT adherence is not an inherent limitation of the test itself but rather a modifiable outcome dependent on the quality and consistency of program-level support [75,76]. Research indicates that sending reminders through mail, automatic calls and involving primary care can help in increasing retention, but sustaining these requires continuous support and infrastructure investment [75,76,77].

### 6.2. mt-sDNA Completion and Adherence

Multitarget stool DNA (mt-sDNA) testing shows higher one-time completion rates which sometimes go beyond 75–85%, especially among people never screened before or those who do not want invasive procedures [33]. The triennial schedule and non-invasive process make it a viable option for patients not ready to commit to annual screening. In an analysis involving one large health system, offering mt-sDNA testing made the patients almost twice as likely to complete screening when compared to the group being offered FIT [33]. Nevertheless, as mt-sDNA is designed for limited use, its success depends on the complete and timely diagnosis and follow-up after a positive result [33].

### 6.3. Colonoscopy After Positive Results

A diagnostic colonoscopy after a positive stool test is one of the most important and decisive factors in the screening process. Unfortunately, follow-up rates are still suboptimal. Some research conducted on both Medicare claims data and the patients of integrated delivery systems indicated that only 42–60% of the individuals who tested positive with FIT had undergone colonoscopy within six months, whereas the rate was 75–85% for those who tested positive with mt-sDNA. This difference is probably less related to the quality of between-the-lines navigation support and more to do with assumed test urgency and the level of patient involvement [73,74,78].

### 6.4. Care Coordination Gaps

Several barriers lead to missed follow-up colonoscopies, such as the absence of centralized navigation, insurance refusals, transport problems, language-related difficulties and the fear of a cancer diagnosis. Systems that combine EMR reminders, nurse navigators and multilingual outreach have much greater follow-up adherence and shorter time to diagnosis. The Veterans Health Administration and Kaiser Permanente are two health systems that have achieved >80% colonoscopy completion rates by coordinating follow-up workflows [79,80,81].

### 6.5. Health Equity and Demographic Performance

Given that screening disparities disproportionately affect racial and ethnic minorities, rural populations, and individuals with limited healthcare access, the comparative performance of FIT and mt-sDNA across demographic subgroups is a critical consideration for real-world implementation.

Several studies have demonstrated that mailed FIT outreach programs achieve higher screening participation among underserved populations, particularly those without established primary care relationships or regular healthcare access. The low cost, simple workflow and minimal logistical requirements of FIT make it particularly well-suited for federally qualified health centers, community health programs and population-level outreach in resource-limited settings.

In contrast, mt-sDNA testing presents logistical barriers that may disproportionately affect vulnerable populations. The requirement for secure shipping, timely sample return within a specified window and centralized laboratory processing creates challenges for individuals with limited transportation, unreliable internet access or low health literacy. Rural populations and those in areas with limited mail infrastructure may face additional difficulties completing the mt-sDNA collection and return process [77].

Data on racial and ethnic disparities in test completion are limited, but emerging evidence suggests differential uptake patterns. Similarly, follow-up colonoscopy completion rates after positive screening tests vary substantially across demographic groups, with lower rates consistently observed among uninsured, non-English speaking and rural populations [79,80,81].

From an equity perspective, the choice between FIT and mt-sDNA should account not only for test sensitivity but also for the accessibility, acceptability and sustainability of the screening pathway within the target population. Programs serving predominantly underserved communities may achieve greater impact through well-supported annual FIT programs with intensive navigation support than through mt-sDNA testing without corresponding infrastructure to address follow-up barriers (Table 3).

## 7. Choosing the Right Test for the Right Patient

The process of test selection should be tailored to individual patient characteristics and healthcare system capabilities. This section synthesizes the evidence presented in Section 3, Section 4, Section 5 and Section 6 into a practical decision-making framework.

### 7.1. Patient-Level Factors

Clinical risk profile. While both FIT and mt-sDNA are recommended for average-risk screening, test selection may be influenced by personal or family history. Patients with a family history of CRC or advanced adenomas may benefit from the higher sensitivity of mt-sDNA for advanced adenomas (42.4% vs. 23.8% for FIT), though colonoscopy remains the preferred modality for individuals meeting high-risk criteria [55,56]. Inappropriate use of mt-sDNA in truly high-risk populations may delay definitive colonoscopic evaluation [18].

Patient preferences and screening behavior. FIT is most suitable for patients who are actively engaged in annual preventive healthcare and comfortable with repeated annual testing. Conversely, mt-sDNA may appeal to patients who prefer less frequent testing or who have shown poor adherence in the past [33]. However, the triennial interval of mt-sDNA requires sustained motivation over a 3-year period, and patients who are unlikely to complete follow-up colonoscopy after a positive result may be better served by annual FIT with intensive navigation support.

Logistical and health literacy considerations. As discussed in Section 6.4, mt-sDNA requires more complex sample handling, secure shipping and timely return within a specified window. Patients with limited transportation, unreliable mail access or low health literacy may face barriers to completing the mt-sDNA process and may achieve better outcomes with the simpler FIT [82,83].

### 7.2. Health System-Level Factors

Outreach and recall infrastructure. FIT achieves optimal effectiveness when delivered within health systems with robust patient reminder systems, automated recall processes and EMR integration. Systems without this infrastructure may observe substantially lower compliance and adherence, as discussed in Section 6.1. In such settings, the logistical simplicity of triennial mt-sDNA may partially compensate for limited outreach capacity [84,85].

Colonoscopy capacity and follow-up coordination. As outlined in Section 6.3, mt-sDNA generates a higher volume of positive results requiring colonoscopy follow-up (13–16% positive rate vs. 4–8% for FIT). Health systems with limited colonoscopy capacity or poor care coordination may struggle to manage this follow-up burden, leading to delays in diagnostic workup and potential for missed or interval cancers. In such setups, FIT may represent a more sustainable screening strategy.

Cost and reimbursement structure. The substantial difference in per-test cost ($20–30 for FIT vs. $500–600 for mt-sDNA) and the program-level cost implications discussed in Section 5.1 must be considered within the context of local reimbursement policies, screening budgets and opportunity costs.

### 7.3. Population-Level Equity Considerations

As discussed in Section 6.4, equity considerations are essential in tailoring test selection to population characteristics. Populations with limited access to primary care, high colonoscopy no-show rates or significant transportation barriers may benefit more from simpler, repeatable FIT outreach models with intensive patient navigation support. Conversely, mt-sDNA may serve as a bridging strategy for previously unscreened populations to initiate screening participation [18].

### 7.4. Shared Decision-Making Framework

Shared decision-making, incorporating discussion of test frequency, sensitivity, specificity and the potential for follow-up colonoscopy, is essential for informed consent and improved screening uptake [18]. Clinicians should present the comparative evidence outlined in this review in accessible terms, acknowledging both the strengths and limitations of each modality. Key discussion points include:

- The trade-off between higher single-test sensitivity (mt-sDNA) and cumulative detection over repeated testing (annual FIT);
- The different false-positive rates and the implications for unnecessary colonoscopy;
- The importance of adherence by subjects to the recommended screening interval for either test;
- The critical role of timely colonoscopy follow-up after any positive result using either test.

## 8. Implications for Oncology Practice

Late-stage diagnosis and poor treatment outcomes are the major consequences of delayed CRC screening. A rising number of oncologists are confronted with patients with diseases that are at an advanced stage owing to missed screening opportunities [5]. Higher attendance and compliance with stool-based screening tests would lower future oncology workload by thwarting late-stage cancers. Intensive cooperation between primary care, oncology and public health is necessary to prevent screening disparities [86]. Screening could also be integrated into the care of cancer survivors and their first-degree family members who are at higher risk, which may contribute to earlier detection among the most vulnerable populations [8,9,10,11,12,13,14,15,16,17,18,19,20,21,22,23,24,25,26,27,28,29,30,31,32,33,34,35,36,37,38,39,40,41,42,43,44,45,46,47,48,49,50,51,52,53,54,55,56,57,58,59,60,61,62,63,64,65,66,67,68,69,70,71,72,73,74,75,76,77,78,79,80].

## 9. Conclusions

A critical finding of this review is that the programmatic implications of the different screening intervals (annual for FIT, triennial for mt-sDNA) are substantial. While mt-sDNA offers higher single-round sensitivity, the cumulative detection rate of three annual FIT rounds over the same 3-year period approaches that of a single mt-sDNA test, particularly when adherence to repeat testing is maintained. Similarly, the cumulative false-positive probability and colonoscopy burden must be evaluated on a per-cycle basis rather than a per-test basis to enable meaningful programmatic comparisons. These temporal dynamics reinforce the central conclusion that test selection should be guided by program-level effectiveness rather than isolated test characteristics.

Strategically, rather than competitively, defining patient goals and system capabilities is critical for framing FIT and mt-sDNA testing models. When both tests are applied with appropriate follow-up and care coordination, they demonstrate value in preventing colorectal cancer. Flexible, patient-centered screening strategies that optimize adherence and resource utilization should be supported by health systems. Stool-based CRC screening will require investments in population outreach, EMR integration and active care navigation to realize its full preventive potential. Real-world comparative effectiveness studies, particularly those involving marginalized populations and modeling the long-term population-level health effects of different screening test implementation strategies, are crucial factors to consider for future research.

## Figures and Tables

**Table 2:** Summary of key advantages and limitations of FIT and mt-sDNA for colorectal cancer screening. Synthesized from: Imperiale, T.F., et al. (2014); Knudsen, A.B., et al. (2016); Fendrick, A.M., et al. (2020) [18,32,50].

Dimension	FIT	mt-sDNA
Cost	Low	High
Scalability	High	Moderate
Single-Test Sensitivity	Moderate	High
False Positive Rate	Low	Higher
Specificity	High	Moderate
Colonoscopy Burden	Lower	Higher

**Table 3:** Follow-up completion and adherence metrics for FIT and mt-sDNA screening programs. Data derived from Halm, E.A., et al. (2025); Gupta, H., et al. (2025); Chubak, J., et al. (2022) [28,31,34,73].

Metric	FIT	mt-sDNA
First-Time Completion Rate	60–70%	75–85%
Repeat Annual Adherence	~40% by year 3	Not applicable
Colonoscopy Completion After Positive Test	~50%	~80%

## Data Availability

No new data were created or analyzed in this study. Data sharing is not applicable to this article.
